# Validity of a Self-Administered Food Frequency Questionnaire for Middle-Aged Urban Cancer Screenees: Comparison With 4-Day Weighed Dietary Records

**DOI:** 10.2188/jea.JE20100173

**Published:** 2011-11-05

**Authors:** Ribeka Takachi, Junko Ishihara, Motoki Iwasaki, Satoko Hosoi, Yuri Ishii, Shizuka Sasazuki, Norie Sawada, Taiki Yamaji, Taichi Shimazu, Manami Inoue, Shoichiro Tsugane

**Affiliations:** 1 Epidemiology and Prevention Division, Research Center for Cancer Prevention and Screening, National Cancer Center, Tokyo, Japan; 2 Department of Community Preventive Medicine, Division of Social and Environmental Medicine, Niigata University Graduate School of Medical and Dental Sciences, Niigata, Japan; 3 Department of Nutrition Management, Sagami Women’s University, Kanagawa, Japan

**Keywords:** dietary assessment, food frequency questionnaire, validity

## Abstract

**Background:**

The validity of estimates of dietary intake calculated using a food frequency questionnaire (FFQ) depends on the specific population. The 138-item FFQ used in the 5-year follow-up survey for the Japan Public Health Center-based Prospective Study was initially developed for and validated in rural residents. However, the validity of estimates based on this FFQ for urban residents, whose diet and lifestyle differ from those of rural residents, has not been clarified. We examined the validity of ranking individuals according to level of dietary consumption, as estimated by this FFQ, among an urban population in Japan.

**Methods:**

Among 896 candidates randomly selected from examinees of cancer screening provided by the National Cancer Center, Japan, 144 participated in the study. In 2007–2008, at an average 2.7 years after cancer screening, participants were asked to respond to the questionnaire and to provide 4-day weighed diet records (4d-DRs) for use as the reference intake. Spearman correlation coefficients (CCs) between the FFQ and 4d-DR estimates were calculated, after correction for intraindividual variation of 4d-DRs.

**Results:**

The median (range) deattenuated CC for men and women was 0.57 (0.23 to 0.89) and 0.47 (0.08 to 0.94), respectively, across 45 nutrients and 0.51 (0.10 to 0.98) and 0.51 (−0.36 to 0.88) for 43 food groups.

**Conclusions:**

Although the FFQ was developed for a rural population, it provided reasonably valid measures of consumption for many nutrients and food groups in middle-aged screenees living in urban areas in Japan.

## INTRODUCTION

Accuracy in measuring individual dietary intake is an important issue in the analysis and evaluation of results from epidemiologic studies of the association between diet and disease. Food frequency questionnaires (FFQs) provide a view of usual food or nutrient intake over time and have been developed and validated in target populations of epidemiologic studies.^1^ Because the foods listed in an FFQ are selected according to their percentage contribution to the total consumption of nutrients among representatives of the target population for whom the FFQ is to be used, they might not necessarily reflect the foods eaten by a different population. Further, accuracy in remembering foods consumed appears to differ by education level and the degree of interest in diet.^1^ The validity of FFQ estimates of dietary intake therefore appears to depend on the specific population.

The FFQ used for the Japan Public Health Center-based Prospective Study 5-year follow-up survey was developed for use with residents of rural cohort areas.^2^ Of these resident, 27% worked in management, clerical, sales, or services, and 21% were employed in the agriculture, forestry, and fisheries sector.^3^ Further, the FFQ was validated among subsamples of these rural residents.^4^^–^^6^ It is therefore unclear whether this FFQ is accurate in estimating dietary intake among Japanese with an urbanized lifestyle. In addition, to our knowledge no such validation study has been restricted to an examination of subjects living in urban and adjacent areas.^7^

To confirm the suitability of this FFQ for use in epidemiologic studies of cancer screenees at the National Cancer Center, such as the participants in the Colorectal Adenoma Study in Tokyo, we evaluated the validity and reproducibility of ranking individuals by levels of dietary consumption—as estimated by this FFQ after minor modification—as a means of assessing dietary intake among middle-aged urban cancer screenees.^8^

## METHODS

### Study setting and participants

The study participants were selected from adults who underwent cancer screening at the Research Center for Cancer Prevention and Screening, National Cancer Center, “Japan from January 2004 through July 2006. Eligibility criteria were age between 40 and 69 years, residence in metropolitan Tokyo, and no previous or present diagnosis of cancer, cardiovascular disease, or diabetes mellitus. Eligible subjects were stratified by sex and age (40–49, 50–59, and 60–69 years) and randomly numbered for recruiting priority.

Among the 896 invited candidates, 187 (response rate: 20.9%) agreed to participate in the study. After excluding those who could not attend the study orientation, 144 participated in the study. As an incentive to participate, participants received a report of their results regarding the consumption of energy and nutrients based on 4-day dietary records, a small gift (an instrument for measuring the salt concentration of soup), and a free invitation to attend a class on healthful cooking. The study was approved by the Institutional Review Board of the National Cancer Center, Tokyo, Japan. All participants provided their written informed consent for participation, at the study orientation.

### Data collection

The reference intake was 4-day weighed diet records (4d-DRs), which were obtained over 4 consecutive days during the period from May 2007 through April 2008. Before the start of data collection, all participants were invited to attend the study orientation, where the 4d-DR procedure was explained by trained dietitians. The self-administered FFQ was first administered during 2004–2006 at the time of cancer screening (FFQ0) and then during 2007–2008 at the orientation session (FFQ1).

### Dietary assessment

The 4d-DR included 3 weekdays and 1 weekend day and was used as the reference method. Food portions were measured by each participant during meal preparation using supplied digital scales and measuring spoons and cups. For foods purchased or consumed outside the home, the participants were instructed to record the approximate quantity of all foods in the meal and/or the names of the product and company. Daily weighed records were faxed to the study office at the Research Center for Cancer Prevention and Screening, National Cancer Center on the morning after completion of that day’s record. Trained dietitians checked the record with the examinee by telephone and coded the foods and weights. Stores and restaurants were asked about the recipes of certain meals eaten outside the home.

The FFQ consisted of 138 food and beverage items and 9 frequency categories, which ranged from almost never to 7 or more times per day (or to 9 glasses per day, for beverages), and asked about the usual consumption of listed foods during the previous year. The food list, which was initially developed for the Japan Public Health Center-based Prospective Study,^2^ was modified for a middle-aged urban population as follows: 11 foods mainly consumed in specific areas (Okinawa and Nagano) or at specific times were excluded (luncheon meats, vivipara, *qing-geng-cai* [bok choy], leaf mustard, bitter gourd, chard, loofah, mugwort, *yushi-tofu* [soft, boiled tofu], calcium beverages, and beta carotene beverages), and 11 foods consumed throughout the year in urban areas were added (beef, stir-fried; chicken, stir-fried; chicken, stew; low-fat milk; Japanese amberjack; Welsh onion; eggplant; edible burdock; *konnyaku* foods [devil’s tongue]; and jam, strawberry or marmalade). Portion size was specified for each food item, using 3 standard sizes: medium (the standard amount), small (50% smaller), and large (50% larger).

Intakes of energy, 45 nutrients, and 43 food groups were calculated using the Standardized Tables of Food Composition, Fifth revised edition^9^^,^^10^ and a specially developed food composition table for isoflavones and lycopene in Japanese foods.^11^^,^^12^ We collapsed the individual food items into 18 predefined food groups according, to the Food Composition Tables, and 25 stream-specific subgroups. The grouping scheme for subgroups, eg, cruciferous vegetables and red meat, was based on the similarity of nutrient profiles or culinary usage among the foods and was somewhat similar to that used in other studies.

### Statistical analysis

The mean intake of each nutrient and food group estimated using the FFQ1 was compared to that estimated using the 4d-DR among the 143 participants who completed both. Percentage differences were calculated for each nutrient and food group by dividing the difference in intake on the FFQ1 from that on the 4d-DR by those using the 4d-DR. To determine the validity of the FFQ, Spearman rank correlation coefficients (CCs) between intake estimates of the FFQ1 and 4d-DR were calculated for crude and energy-adjusted values. Regression coefficients between nutrient intakes according to the FFQ1 and 4d-DR were calculated for energy-adjusted values to examine the degree of attenuation in a diet–disease association in a hypothetical study using the FFQ.^1^ A residual model was used for energy adjustment.^1^ We corrected the observed CCs for the attenuating effect of random intraindividual error from the usual intake of each energy and nutrient and each food group. The deattenuated value was corrected using the ratios of the within- to between-individual variances based on the 4-day DRs according to the following formula:

$${\text{deattenuated CC}}_{x}={\text{en-CC}}_{x}*\text{SQRT}(1+\lambda_{x}/n),$$

where the observed en-CC_x_ is the correlation in energy-adjusted value for nutrient x, λ_x_ is the ratio of within- to between-individual variance, and n is the number of dietary records (4 days).^1^ To measure the validity of categorization, we computed the number of participants classified into the same, adjacent, and extreme categories by joint classification according to both quintiles using the FFQ1 and the 4d-DR. For reproducibility, CCs between the FFQ1 and FFQ0 were calculated for crude and energy-adjusted values for the 144 participants who completed both FFQs. We confirmed the cumulative percentage among the top 20 foods for energy, because food variety was important in confirming the extent to which the list of FFQ items could be covered. Percentages of the sum of energy by individual foods eaten to total energy during the 4 days were also calculated. All analyses were performed using SAS Version 9.1 (SAS Institute Inc., Cary, NC).

## RESULTS

### Participants in the validation study

Age distribution (40s, 50s, 60s) at recruitment (2004–2006) was *n* = 11, 29, and 29, respectively, for men and *n* = 16, 30, and 29 for women. Mean body mass index (standard deviation) for men and women was 23.5 (2.5) and 21.5 (2.5), respectively. Overall, 51% of the participants were employed in management, clerical, sales, or services, and 2% worked in agriculture, forestry, or fisheries.

### Mean intakes and FFQ validity

Table 1 shows daily intakes of energy and 45 nutrients, as assessed by 4d-DR and FFQ1, percentage differences between FFQ1 and the 4d-DR, and their correlations among men and women. Although estimated intake levels for energy were very similar between the 2 methods (difference: −6% for men, 2% for women), the percentage difference in nutrient intake between the 4d-DR and FFQ1 varied from −35% and −20% for beta-carotene to +99% and +198% for cryptoxanthin in men and women, respectively. The CCs of the crude values varied from 0.12 for retinol equivalents to 0.71 for daidzein in men and from 0.10 for polyunsaturated fatty acid to 0.57 for vitamin K in women. The median across the 45 nutrients was 0.43 for both men and women. After energy adjustment and deattenuation, the median CC improved to 0.57 in men and 0.47 in women. The regression coefficient for nutrient intake varied from 0.16 for retinol equivalents to 0.61 for copper in men and from 0.05 for cryptoxanthin to 0.63 for pantothenic acid in women (data not shown).

**Table 1.** Energy and nutrient intakes according to food frequency questionnaire 1 (FFQ1), percentage difference between FFQ1 and 4-day diet record (DR), and their correlations in men and women

	Men (*n* = 69)						Women (*n* = 74)					
		
	4-day DR	FFQ1^a^	%^b^	Correlation coefficient^c^			4-day DR	FFQ1^a^	%^b^	Correlation coefficient^c^		
						
	Mean ± SD	Mean ± SD		Crude	Energy- adjusted	Deatte- nuated^d^	Mean ± SD	Mean ± SD		Crude	Energy- adjusted	Deatte- nuated^d^
Energy (kcal)	2271 ± 426	2141 ± 737	−6	0.48	—	0.53	1842 ± 298	1875 ± 733	2	0.29	—	0.34
Protein (g)	89.2 ± 15.6	76.2 ± 32.3	−15	0.31	0.55	0.67	75.0 ± 13.6	70.5 ± 32	−6	0.56	0.39	0.47
Total fat (g)	64.6 ± 14.3	64.6 ± 33.2	0	0.27	0.30	0.42	57.8 ± 16.3	63.0 ± 32.9	9	0.22	0.28	0.35
SFA (g)	18.12 ± 4.85	20.21 ± 11.23	12	0.27	0.29	0.44	16.82 ± 5.84	20.04 ± 12.21	19	0.37	0.34	0.41
MUFA (g)	22.63 ± 6.56	22.79 ± 13.15	1	0.31	0.28	0.38	20.34 ± 7.06	22.59 ± 12.41	11	0.26	0.39	0.49
PUFA (g)	14.62 ± 3.21	13.38 ± 6.73	−8	0.53	0.53	0.72	12.33 ± 2.99	12.4 ± 6.01	1	0.10	0.24	0.38
n-3 PUFA (g)	3.10 ± 1.05	2.58 ± 1.59	−17	0.26	0.34	0.56	2.48 ± 0.91	2.35 ± 1.28	−5	0.34	0.28	0.68
n-6 PUFA (g)	11.45 ± 2.74	10.73 ± 5.43	−6	0.58	0.53	0.76	9.79 ± 2.51	9.97 ± 4.81	2	0.11	0.31	0.47
Cholesterol (mg)	367 ± 132	303 ± 278	−18	0.31	0.39	0.51	333 ± 117	271 ± 168	−19	0.35	0.28	0.38
Carbohydrate (g)	301 ± 72.9	270.8 ± 99.2	−10	0.58	0.52	0.56	245.2 ± 46.9	245.7 ± 84.4	0	0.25	0.39	0.43

Total dietary fiber (g)	20.3 ± 6.3	14.1 ± 6.6	−31	0.55	0.61	0.67	18.0 ± 4.6	15.3 ± 7.4	−15	0.44	0.46	0.53
Water soluble (g)	4.7 ± 1.7	3.6 ± 1.9	−23	0.53	0.59	0.65	4.1 ± 1.2	3.8 ± 1.9	−9	0.44	0.48	0.56
Water insoluble (g)	14.3 ± 4.6	9.9 ± 4.6	−31	0.55	0.64	0.71	13.0 ± 3.4	10.9 ± 5.3	−16	0.43	0.38	0.44
Sodium (mg)	4728 ± 1745	4269 ± 2312	−10	0.44	0.42	0.45	3943 ± 944	3920 ± 1953	−1	0.33	0.39	0.47
Salt equivalent (g)	11.9 ± 4.4	10.8 ± 5.9	−9	0.44	0.39	0.42	9.9 ± 2.4	9.9 ± 4.9	0	0.33	0.38	0.46
Potassium (mg)	3695 ± 983	3072 ± 1208	−17	0.37	0.60	0.65	3204 ± 708	2992 ± 1318	−7	0.48	0.62	0.70
Calcium (mg)	707 ± 234	665 ± 423	−6	0.48	0.58	0.64	637 ± 204	657 ± 469	3	0.55	0.55	0.61
Magnesium (mg)	393 ± 112	317 ± 117	−19	0.43	0.53	0.58	323 ± 64	293 ± 125	−9	0.43	0.45	0.54
Phosphorus (mg)	1395 ± 296	1221 ± 512	−12	0.38	0.57	0.65	1183 ± 227	1144 ± 569	−3	0.55	0.40	0.47
Iron (mg)	11.2 ± 3.2	9.6 ± 3.8	−15	0.45	0.62	0.68	9.3 ± 2	8.8 ± 3.5	−6	0.46	0.44	0.55
Zinc (mg)	10.0 ± 2.2	8.8 ± 3.5	−12	0.40	0.53	0.65	8.7 ± 1.8	7.8 ± 3.2	−10	0.49	0.26	0.34
Copper (mg)	1.59 ± 0.41	1.35 ± 0.54	−15	0.59	0.67	0.74	1.31 ± 0.26	1.23 ± 0.47	−6	0.35	0.40	0.49
Manganese (mg)	5.03 ± 2.7	4.22 ± 1.75	−16	0.54	0.41	0.44	3.93 ± 1.31	4.35 ± 2.13	11	0.41	0.37	0.41

Retinol (µg)	318 ± 379	364 ± 308	14	0.21	0.32	0.56	348 ± 528	361 ± 274	4	0.13	0.11	0.16
Retinol Eq (µg)	749 ± 433	678 ± 383	−10	0.12	0.15	0.23	782 ± 560	754 ± 412	−4	0.35	0.24	0.33
α-carotene (µg)	542 ± 381	474 ± 387	−13	0.38	0.37	0.50	667 ± 534	632 ± 736	−5	0.51	0.53	0.78
β-carotene (µg)	4580 ± 2697	2960 ± 1854	−35	0.34	0.36	0.49	4588 ± 2281	3658 ± 2751	−20	0.54	0.53	0.70
Cryptoxanthin (µg)	539 ± 1148	1071 ± 1262	99	0.50	0.52	0.55	482 ± 668	1439 ± 1656	198	0.15	0.07	0.08
Lycopene (mg)	6583 ± 7892	4888 ± 7441	−26	0.48	0.45	0.52	4456 ± 5151	4319 ± 5617	−3	0.23	0.33	0.40
β-carotene Eq (µg)	5152 ± 2860	3731 ± 2289	−28	0.40	0.39	0.52	5194 ± 2625	4693 ± 3391	−10	0.54	0.49	0.62
Vitamin D (µg)	11.3 ± 6.5	7.9 ± 5.8	−30	0.47	0.52	0.88	9.9 ± 6.1	8.1 ± 6.2	−18	0.34	0.22	0.37
α-tocopherol (mg)	9.8 ± 3.0	8.1 ± 4.2	−17	0.26	0.41	0.48	8.6 ± 2.5	8.1 ± 4.3	−6	0.27	0.42	0.51
β-tocopherol (mg)	0.4 ± 0.1	0.4 ± 0.2	0	0.34	0.30	0.48	0.3 ± 0.1	0.4 ± 0.2	17	0.14	0.21	0.33
γ-tocopherol (mg)	13 ± 4	12 ± 6.8	−8	0.53	0.47	0.69	11.1 ± 3.5	10.9 ± 5.3	−1	0.10	0.22	0.33
δ-tocopherol (mg)	3.4 ± 1.4	3 ± 2.1	−10	0.69	0.68	0.89	2.7 ± 0.9	2.6 ± 1.2	−4	0.18	0.25	0.51
Vitamin K (µg)	345 ± 194	303 ± 306	−12	0.64	0.67	0.79	290 ± 108	270 ± 133	−7	0.57	0.61	0.94
Vitamin B_1_ (mg)	1.21 ± 0.38	1.01 ± 0.43	−17	0.23	0.47	0.54	1.05 ± 0.28	0.99 ± 0.45	−5	0.44	0.35	0.42
Vitamin B_2_ (mg)	1.71 ± 0.55	1.66 ± 0.79	−3	0.27	0.38	0.42	1.47 ± 0.37	1.58 ± 0.8	8	0.47	0.46	0.53
Niacin (mg)	24.2 ± 7.3	20.3 ± 8.6	−16	0.38	0.36	0.44	19.8 ± 5.2	19.0 ± 8.4	−4	0.44	0.26	0.32
Vitamin B_6_ (mg)	1.91 ± 0.55	1.57 ± 0.6	−18	0.38	0.39	0.44	1.53 ± 0.4	1.45 ± 0.63	−5	0.46	0.49	0.57
Vitamin B_12_ (µg)	10.8 ± 5.6	7.9 ± 5.1	−27	0.13	0.30	0.57	8.6 ± 4.6	7.2 ± 5.1	−16	0.46	0.36	0.67
Folate (µg)	512 ± 188	418 ± 176	−18	0.48	0.60	0.66	449 ± 124	433 ± 194	−3	0.48	0.35	0.41
Pantothenic acid (mg)	8.02 ± 1.9	7.66 ± 3.5	−4	0.41	0.58	0.67	6.83 ± 1.5	7.09 ± 3.15	4	0.53	0.57	0.66
Vitamin C (mg)	178 ± 82	136 ± 83	−24	0.62	0.67	0.73	156 ± 62	163 ± 96	4	0.45	0.45	0.51

Daidzein (mg)	17.14 ± 9.78	20.39 ± 20.45	19	0.71	0.66	0.84	12.81 ± 7.28	14.98 ± 8.08	17	0.49	0.49	0.79
Genistein (mg)	28.6 ± 16.27	34.13 ± 35.71	19	0.69	0.64	0.81	21.87 ± 12.3	24.84 ± 13.7	14	0.46	0.47	0.75

**MEDIAN**				**0.43**	**0.52**	**0.57**				**0.43**	**0.39**	**0.47**

Abbreviations: SD, standard deviation; SFA, saturated fatty acid; MUFA, monounsaturated fatty acid; PUFA, polyunsaturated fatty acid; Eq, equivalent.

^a^Intakes based on second FFQ, conducted in 2007–2008. ^b^Percentage differences: (FFQ1 − DR)/DR * 100 (%). ^c^Spearman’s rank correlation coefficients based on crude and energy-adjusted values. For men, *r* ≥ 0.24 = *P* < 0.05, *r* ≥ 0.31 = *P* < 0.01, *r* ≥ 0.39 = *P* < 0.001. For women, *r* ≥ 0.23 = *P* < 0.05, *r* ≥ 0.30 = *P* < 0.01, *r* ≥ 0.38 = *P* ≤ 0.001. ^d^Deattenuated CC_x_ = observed CC_x_ * SQRT(1 + λ_x_/n), where λ_x_ is the ratio of within- to between-individual variance for nutrient x, and n is number of dietary records; observed CCs were based on energy-adjusted values other than energy intake.

Table 2 shows daily intakes of 43 food groups assessed by the 4d-DR and FFQ1, the percentage difference between FFQ1 and 4d-DR, and their correlations among men and women. The percent difference in intakes between the 4d-DR and FFQ1 varied from −83% and −86% for seasonings and spices in men and women, respectively, to +111% for other cereals in men and +153% for citrus fruit in women. The CCs of the crude values varied from 0.04 and −0.28 for seasonings to 0.81 and 0.82 for coffee in men and women, respectively. The medians across 43 food groups for men and women were 0.45 and 0.35, respectively. After energy adjustment and deattenuation, the median CC slightly improved to 0.51 (varying from 0.10 for seasonings to 0.98 for noodles) in men and 0.51 (varying from −0.36 for seasonings to 0.88 for coffee) in women.

**Table 2.** Food-group intakes according to food frequency questionnaire 1 (FFQ1), percentage difference between FFQ1 and 4-day diet record (DR), and their correlations in men and women

	Men (*n* = 69)						Women (*n* = 74)					
		
	4-day DR	FFQ1^a^	%^b^	Correlation coefficient^c^			4-day DR	FFQ1^a^	%^b^	Correlation coefficient^c^		
						
	Mean ± SD (g)	Mean ± SD (g)		Crude	Energy- adjusted	Deatte- nuated^d^	Mean ± SD (g)	Mean ± SD (g)		Crude	Energy- adjusted	Deatte- nuated^d^
Cereals	447 ± 173	510 ± 215	14	0.67	0.45	0.51	332 ± 78	425 ± 153	28	0.29	0.33	0.41
Rice	306 ± 169	351 ± 173	15	0.72	0.42	0.51	210 ± 85	259 ± 110	24	0.49	0.43	0.59
Bread	47 ± 38	40 ± 57	−13	0.66	0.67	0.80	42 ± 27	53 ± 77	26	0.60	0.68	0.87
Noodles	85 ± 71	97 ± 88	15	0.53	0.52	0.98	72 ± 49	95 ± 72	33	0.39	0.42	—
Other cereals	10 ± 11	21 ± 34	111	0.15	0.15	0.19	8 ± 11	17 ± 24	103	0.26	0.26	0.33
Potatoes and starches	46 ± 33	27 ± 21	−41	0.29	0.32	0.49	44 ± 32	38 ± 29	−13	0.09	0.25	0.39
Sugar	9 ± 8	2 ± 4	−80	0.36	0.25	0.30	8 ± 8	1 ± 4	−82	0.07	0.06	0.07
Pulses	102 ± 106	97 ± 144	−6	0.59	0.53	0.66	72 ± 44	67 ± 44	−6	0.27	0.30	0.45
Nuts and seeds	7 ± 11	3 ± 4	−61	0.31	0.30	0.40	5 ± 7	3 ± 9	−33	0.01	−0.06	−0.09
Vegetables	403 ± 180	218 ± 145	−46	0.48	0.48	0.55	354 ± 125	245 ± 175	−31	0.39	0.45	0.52
Green and yellow vegetables	194 ± 134	110 ± 90	−43	0.43	0.47	0.59	170 ± 86	114 ± 87	−33	0.38	0.41	0.57
White vegetables	209 ± 102	108 ± 96	−48	0.53	0.50	0.68	184 ± 75	131 ± 128	−29	0.39	0.41	0.57
Pickled vegetables	21 ± 51	15 ± 21	−32	0.43	0.37	0.42	18 ± 21	21 ± 50	21	0.32	0.34	0.45
Cruciferous vegetables	91 ± 59	54 ± 68	−41	0.63	0.64	0.82	87 ± 64	55 ± 41	−37	0.46	0.45	0.54
Green, leafy vegetable	43 ± 43	20 ± 20	−54	0.33	0.28	0.37	38 ± 23	21 ± 14	−43	0.26	0.29	0.41
Yellow vegetables	128 ± 113	78 ± 83	−39	0.49	0.52	0.63	105 ± 73	79 ± 76	−25	0.36	0.42	0.51
Other vegetables	121 ± 73	54 ± 38	−56	0.31	0.36	0.51	109 ± 55	71 ± 73	−35	0.34	0.37	0.56
Fruits	193 ± 160	209 ± 184	8	0.60	0.64	0.69	184 ± 113	255 ± 196	38	0.40	0.55	0.63
Citrus fruit	49 ± 75	81 ± 88	67	0.46	0.46	0.51	43 ± 49	109 ± 139	153	0.23	0.18	0.20
Other fruit	143 ± 126	126 ± 108	−12	0.54	0.57	0.75	140 ± 104	144 ± 103	3	0.31	0.49	0.85
Fungi	18 ± 17	11 ± 11	−36	0.48	0.48	0.57	23 ± 22	14 ± 12	−38	0.42	0.38	0.46
Algae	15 ± 22	8 ± 7	−43	0.18	0.17	0.22	10 ± 10	9 ± 8	−9	0.35	0.32	0.47
Fish and shellfish	115 ± 53	78 ± 66	−32	0.40	0.47	0.69	89 ± 40	73 ± 60	−18	0.44	0.35	0.57
Meats	72 ± 43	62 ± 57	−15	0.43	0.48	0.70	65 ± 38	55 ± 35	−17	0.35	0.26	0.36
Processed meat	13 ± 16	6 ± 8	−52	0.46	0.45	0.63	13 ± 15	7 ± 7	−48	0.30	0.33	0.47
Red meat	40 ± 30	42 ± 43	5	0.36	0.41	0.74	36 ± 29	32 ± 23	−11	0.45	0.36	0.53
Poultry	19 ± 26	13 ± 18	−30	0.25	0.25	0.38	16 ± 19	15 ± 13	−5	0.27	0.22	0.36
Eggs	36 ± 23	32 ± 55	−11	0.50	0.46	0.67	33 ± 19	25 ± 30	−24	0.35	0.35	0.53
Milk and dairy products	176 ± 147	275 ± 305	56	0.62	0.58	0.66	174 ± 110	257 ± 337	48	0.70	0.62	0.76
High-fat milk	87 ± 96	99 ± 157	13	0.47	0.44	0.50	95 ± 91	120 ± 201	26	0.64	0.59	0.69
Low-fat milk	89 ± 137	177 ± 286	98	0.62	0.56	0.60	79 ± 83	137 ± 207	74	0.68	0.61	0.70
Fats and oils	11 ± 6	12 ± 8	12	0.40	0.35	0.45	10 ± 6	12 ± 8	24	0.38	0.52	0.73
Butter	2 ± 2	1 ± 2	−49	0.32	0.34	0.50	2 ± 2	1 ± 4	−12	0.35	0.35	0.56
Margarine and oils	9 ± 5	11 ± 8	25	0.31	0.26	0.35	9 ± 6	11 ± 6	31	0.29	0.42	0.57
Confectionaries	29 ± 28	23 ± 32	−19	0.28	0.37	0.45	37 ± 30	37 ± 46	1	0.34	0.32	0.43
Japanese confectionery	11 ± 15	8 ± 15	−29	0.21	0.24	0.33	15 ± 23	15 ± 20	−1	0.09	0.04	0.05
Western confectionery	18 ± 24	15 ± 21	−13	0.34	0.41	0.50	21 ± 21	22 ± 32	2	0.26	0.24	0.32
Alcoholic beverages	219 ± 276	263 ± 281	20	0.80	0.80	0.88	76 ± 151	90 ± 187	19	0.65	0.57	0.60
Nonalcoholic beverages	749 ± 772	863 ± 699	15	0.45	0.37	0.40	617 ± 434	888 ± 621	44	0.33	0.33	0.35
Green tea	386 ± 738	519 ± 427	35	0.68	0.67	0.72	246 ± 220	603 ± 560	145	0.46	0.42	0.45
Coffee	176 ± 204	199 ± 281	13	0.81	0.80	0.84	167 ± 175	157 ± 155	−6	0.82	0.82	0.88
Other beverage	210 ± 260	144 ± 358	−31	0.43	0.45	0.49	268 ± 359	128 ± 190	−52	0.31	0.32	0.35
Seasonings and spices	138 ± 100	23 ± 15	−83	0.04	0.08	0.10	142 ± 111	20 ± 14	−86	−0.28	−0.31	−0.36

**MEDIAN**				**0.45**	**0.45**	**0.51**				**0.35**	**0.35**	**0.51**

Abbreviation: SD, standard deviation.

^a^Intakes based on second FFQ, conducted in 2007–2008. ^b^Percentage differences: (FFQ1 − DR)/DR * 100 (%). ^c^Spearman’s rank correlation coefficients based on crude and energy-adjusted values. For men, *r* ≥ 0.24 = *P* < 0.05, *r* ≥ 0.31 = *P* < 0.01, *r* ≥ 0.39 = *P* < 0.001. For women, *r* ≥ 0.23 = *P* < 0.05, *r* ≥ 0.30 = *P* < 0.01, *r* ≥ 0.38 = *P* ≤ 0.001. ^d^Deattenuated CC_x_ = observed CC_x_ * SQRT(1 + λ_x_/n), where λ_x_ is the ratio of within- to between-individual variance for nutrient x, and n is number of dietary records; observed CCs were based on energy-adjusted values other than energy intake.

—: not applicable for calculation.

### Joint classification by quintile

We conducted further analysis to compare FFQ1 with the 4d-DR based on joint classification by quintile. Most nutrients and food groups were classified into the opposite extreme categories by 5% or less of men or women, with a corresponding median value for men and women of 1% and 3%, respectively, for nutrients, and of 3% and 3%, respectively, for food groups (Supplemental Tables 1 and 2). In contrast, retinol for men and women showed a relatively high percentage of extreme categories by joint classification (6% and 12%, respectively) and a relatively low CC (0.32 and 0.11, respectively) and regression coefficient (0.18 and 0.15, respectively). Further, cryptoxanthin for women showed a relatively low percentage of the same and adjacent categories (53%) and a relatively low CC (0.07) and regression coefficient (0.05).

### Reproducibility

We also examined the reproducibility of dietary intake estimated by 2 identical FFQs (FFQ0 and FFQ1) administered at an average interval of 2.7 years (range 1.3–4.0 years). CCs for nutrient intakes in the crude values varied from 0.54 for retinol to 0.80 for phosphorus (median *r* = 0.70) in men and from 0.48 for cholesterol and 0.72 for vitamin C (median *r* = 0.61) in women. With regard to the food groups, CC in the crude values varied from 0.35 for other cereals to 0.75 for coffee (median *r* = 0.64) in men and from 0.48 for red meat and 0.80 for coffee (median *r* = 0.63) in women (Supplemental Tables 3 and 4).

### Percentage contributions of the top 20 foods to total energy

Finally, we conducted an additional analysis of the cumulative percentage contributions of the top 20 foods for energy, based on the 4d-DRs, to assess the foods listed in the FFQ. The cumulative percentage of the top 20 foods for energy was 44.0% and 41.0% for men and women, respectively (Supplemental Table 5).

## DISCUSSION

We examined the validity of ranking middle-aged urban-dwelling cancer screenees in Japan by level of dietary intake using an FFQ, with 4-day DR data as the reference method. The FFQ was initially developed and validated in rural populations. As compared with reference intakes, differences in mean absolute consumption based on the FFQ varied and tended to be underestimated. However, using the FFQ, dietary assessment of many nutrients and food groups showed moderate validity and reproducibility in ranking urban residents, whose diet and lifestyle might differ from those of rural residents.

In comparison with 4d-DRs corrected for intraindividual variance, for most nutrients, the validity of the FFQ was similar to or better than that observed in a comparison with 28-day weighed diet records among the rural residents for which the FFQ was developed.^6^ In that initial validation study, median CCs for energy and 45 nutrients were 0.43 and 0.39 for men and women, respectively, and 0.38 and 0.32 for 19 main food groups. Evaluation of diet might be complicated by the apparently wider variety of foods eaten by urban as compared with rural residents in Japan (percent energy from cereal areas among the former was less than that among the latter^13^), as has been seen in China^14^ and Morocco,^15^ although we saw no large difference in the validity of intakes, as estimated by the FFQ, between urban and rural populations in the present study.

Wakai^7^ reviewed 21 validation studies of FFQs developed in Japan and reported a median CC for energy intake of 0.46 (range 0.20 to 0.87) and a median CC among the 21 studies ranging from 0.22 (n-6 PUFA) to 0.58 (calcium) for energy and 24 nutrients. As compared with the median CCs among the 21 studies for energy and 24 nutrients and 17 food groups, the CCs for the many nutrients and food groups evaluated in the present study were not substantially different or higher.^7^ Attenuation caused by measurement error may be unavoidable in studies that use FFQs to investigate diet–disease associations. For example, based on a true relative risk of 2.0, if the regression coefficient of intakes according to an FFQ and DR varies from 0.6 to 0.2, the corresponding relative risk is further attenuated from 1.52 to 1.15.^1^ A similar attenuation might be unavoidable in any examination that uses the present FFQ to assess diet–disease associations. Further investigation will be needed to examine the effects of measurement error on diet–disease associations in an actual dataset.

The CC for energy intake among women in this study (deattenuated CC: *r* = 0.34) was lower than the median of 21 previous studies. Further, the CCs of intakes based on the FFQ appeared to be lower in women than in men for most of the energy and nutrients examined (median deattenuated CC: 0.57 and 0.47 for men and women, respectively). This lower correlation in women than men has been previously observed in Japanese and Western populations.^7^^,^^16^ Sex differences in validity might be partly due to disparities in the ease of response to the structured questionnaire that result from differences between men and women in their interest in dietary habits.^4^ Moreover, we also found that the cumulative percentage among the top 20 foods for energy was lower for women than for men and that it was also lower than among subjects during the development of the initial FFQ (men: 63.9%, women: 56.3%).^17^ These results suggest that the lower validity for energy intake among women is partly attributable to a lower contribution to energy by individual foods in women than in men, as was seen among subjects during the development of the initial FFQ.

Our study has several potential limitations. First, the response rate was not necessarily high, although the participants were randomly chosen and recruited from among cancer screenees. Selection bias, eg, a higher proportion of health-conscious subjects than in the actual population, was likely present, and thus the possibility of overestimating the validity of the FFQ cannot be ruled out. This response rate is nevertheless reasonable considering the burden posed by studies such as this. Second, reference intakes were based on 4-day values, versus the 28-day values used for the initial validation study of the FFQ.^4^^–^^6^ A simple comparison of CCs might have been difficult, even though the present CCs were corrected for intraindividual variance. Moreover, although the dietary records were completed on consecutive days (ie, in the same season), the FFQ inquired about the previous year. In addition, responses to the FFQ might have depended on the season,^18^ and FFQ1 was conducted in the season during which the dietary record was done. Thus, the possibility that validity might have been overestimated cannot be ruled out, especially for seasonal foods such as fruit and vegetables. Third, in the examination of reproducibility, we were unable to consider the “true” change in diet. Although we would have liked to examine the effects of random variation in response to the FFQ, the effects of such variation and the “true” change of diet could not be readily separated, and both might have attenuated the reproducibility of the FFQ.^1^ Therefore, the reproducibility of this FFQ (in random variation in response) might have been underestimated.

In general, the advantages of FFQ-based dietary assessment are that the burden on participants is not heavy, an interviewer is unnecessary, costs are relatively low,^19^ and the long-term diet can be ranked. In the present study, too, the median percentages of extreme categories based on joint classification by quintile between FFQ and DR for nutrients and food groups were 1% and 3%, indicating that this FFQ is suitable for the ranking of individuals with regard to intakes of many nutrients and food groups in large-scale studies of urban populations. However, some nutrient and food group intakes estimated by this FFQ showed relatively low CCs and regression coefficients; thus, any application of this FFQ to the examination of diet–disease associations, such as investigations of retinol and cryptoxanthin, must carefully address the problem of classification.

In conclusion, these results indicate that the present FFQ, which was initially developed for rural populations, provides reasonably valid measures in ranking middle-aged cancer screenees in urban areas in Japan according to level of consumption of many nutrients and food groups.

**Supplementary Table 1.** Comparison of food frequency questionnaire 1 (FFQ1) with 4-day diet record for energy-adjusted nutrients, based on joint classification by quintile (%)

	Men (*n* = 69)			Women (*n* = 74)		
		
	Same category	Same and adjacent category	Extreme category	Same category	Same and adjacent category	Extreme category
Energy	35	71	1^a^	28	64	5^a^
Protein	35	77	1	23	60	1
Total fat	28	61	1	31	70	4
SFA	35	65	6	26	65	5
MUFA	22	59	4	31	68	0
PUFA	30	67	0	28	62	4
n-3 PUFA	26	59	3	27	58	3
n-6 PUFA	38	73	0	26	66	5
Cholesterol	25	67	1	28	62	4
Carbohydrate	44	70	1	30	73	4

Total dietary fiber	39	78	1	26	69	1
Water soluble	35	80	1	31	70	1
Water insoluble	33	84	0	24	64	1

Sodium	36	68	3	32	57	1
Salt equivalent	36	68	3	27	61	1
Potassium	38	75	0	39	78	1
Calcium	28	73	0	30	68	0
Magnesium	39	73	0	37	65	1
Phosphorus	35	77	0	35	70	1
Iron	36	80	1	31	72	3
Zinc	38	74	1	23	61	3
Copper	36	80	0	24	65	1
Manganese	28	67	3	31	69	4

Retinol	33	62	6	23	62	12
Retinol Eq	28	62	9	26	62	4
α-carotene	38	68	3	37	70	0
β-carotene	33	65	6	35	70	0
Cryptoxanthin	33	78	3	18	53	4
Lycopene	38	75	4	31	70	5
β-carotene Eq	33	67	4	28	72	1
Vitamin D	36	75	1	22	58	3
α-tocopherol	29	61	1	22	69	3
β-tocopherol	20	67	4	19	57	4
γ-tocopherol	28	68	3	20	68	8
δ-tocopherol	42	80	0	18	68	4
Vitamin K	32	83	0	27	73	0
Vitamin B_1_	29	74	1	32	64	3
Vitamin B_2_	30	65	4	26	70	0
Niacin	30	65	3	27	64	5
Vitamin B_6_	33	67	1	41	69	0
Vitamin B_12_	22	65	4	30	65	3
Folate	32	70	0	26	66	4
Pantothenic acid	45	78	1	42	73	0
Vitamin C	39	87	0	26	68	0

Daidzein	30	81	0	27	76	1
Genistein	32	80	0	31	70	1

**MEDIAN**	**33**	**70**	**1**	**28**	**68**	**3**

Abbreviations: SFA, saturated fatty acid; MUFA, monounsaturated fatty acid; PUFA, polyunsaturated fatty acid; Eq, equivalent.

^a^Joint classification for energy intake was calculated by using crude values.

**Supplementary Table 2.** Comparison of food frequency questionnaire 1 (FFQ1) with 4-day diet record for energy-adjusted food groups based on joint classification by quintile (%)

	Men (*n* = 69)			Women (*n* = 74)		
		
	Same category	Same and adjacent category	Extreme category	Same category	Same and adjacent category	Extreme category
Cereals	26	70	0	32	68	4
Rice	30	71	3	42	72	3
Bread	30	80	0	41	76	0
Noodles	29	68	0	37	72	5
Other cereals	19	52	4	24	55	5
Potatoes and starches	30	67	1	20	64	3
Sugar	26	57	3	16	55	7
Pulses	38	74	3	30	62	3
Nuts and seeds	26	58	0	15	54	10
Vegetables	26	70	1	27	73	3

Green and yellow vegetables	45	68	4	27	70	0
White vegetables	25	77	1	27	68	1
Pickled vegetables	30	68	4	30	68	5

Cruciferous vegetables	42	80	1	31	64	1
Green, leafy vegetable	28	67	6	27	61	1
Yellow vegetables	30	77	3	27	73	1
Other vegetables	19	64	1	28	65	4
Fruits	49	81	1	38	73	0
Citrus fruit	36	77	3	23	60	5
Other fruit	36	77	1	30	69	1
Fungi	33	71	3	24	62	3
Algae	33	58	6	22	64	3
Fish and shellfish	28	71	1	23	61	3

Meats	38	78	6	28	66	7
Processed meat	28	67	1	32	72	3
Red meat	29	71	6	32	62	1
Poultry	25	61	3	28	54	5
Eggs	36	74	1	26	61	3
Milk and dairy products	41	78	3	35	78	0
High-fat milk	41	67	4	42	87	3
Low-fat milk	36	78	3	42	82	3
Fats and oils	30	61	4	39	72	0
Butter	32	64	6	34	70	5
Margarine and oils	29	61	4	34	66	1
Confectionaries	20	67	1	28	62	3
Japanese confectionery	22	64	4	14	55	5
Western confectionery	32	65	0	24	60	5
Alcoholic beverages	46	91	0	42	72	0
Nonalcoholic beverages	26	64	1	22	65	3
Green tea	48	80	0	27	65	0
Coffee	45	93	0	50	91	0
Other beverage	29	68	0	26	65	4
Seasonings and spices	16	49	4	16	42	12

**MEDIAN**	**30**	**68**	**3**	**28**	**65**	**3**

**Supplementary Table 3.** Spearman rank correlation coefficients between 2 food frequency questionnaires, administered at an average interval 2.7 years, for estimated nutrient intakes

	Men (*n* = 69)		Women (*n* = 75)	
		
	Crude	Energy- adjusted	Crude	Energy- adjusted
Energy	0.72	—	0.59	—
Protein	0.76	0.65	0.59	0.55
Total fat	0.73	0.51	0.62	0.40
SFA	0.75	0.54	0.66	0.55
MUFA	0.71	0.47	0.62	0.41
PUFA	0.68	0.62	0.54	0.44
n-3 PUFA	0.64	0.52	0.63	0.59
n-6 PUFA	0.68	0.59	0.52	0.42
Cholesterol	0.76	0.50	0.48	0.46
Carbohydrate	0.65	0.77	0.57	0.43

Total dietary fiber	0.70	0.74	0.62	0.66
Water soluble	0.65	0.65	0.62	0.62
Water insoluble	0.70	0.75	0.64	0.64

Sodium	0.71	0.52	0.66	0.58
Salt equivalent	0.71	0.52	0.66	0.59
Potassium	0.73	0.74	0.65	0.76
Calcium	0.77	0.72	0.62	0.56
Magnesium	0.73	0.75	0.61	0.74
Phosphorus	0.80	0.74	0.61	0.51
Iron	0.70	0.66	0.61	0.69
Zinc	0.71	0.65	0.58	0.67
Copper	0.65	0.69	0.59	0.70
Manganese	0.72	0.75	0.69	0.70

Retinol	0.54	0.39	0.49	0.48
Retinol Eq	0.61	0.45	0.53	0.44
α-carotene	0.65	0.60	0.68	0.63
β-carotene	0.68	0.64	0.68	0.67
Cryptoxanthin	0.68	0.64	0.64	0.72
Lycopene	0.59	0.52	0.49	0.37
β-carotene Eq	0.68	0.64	0.69	0.67
Vitamin D	0.63	0.43	0.67	0.54
α-tocopherol	0.63	0.53	0.58	0.58
β-tocopherol	0.67	0.54	0.52	0.41
γ-tocopherol	0.64	0.51	0.51	0.46
δ-tocopherol	0.68	0.64	0.59	0.58
Vitamin K	0.65	0.67	0.55	0.58
Vitamin B_1_	0.74	0.61	0.59	0.51
Vitamin B_2_	0.74	0.62	0.67	0.67
Niacin	0.71	0.50	0.67	0.55
Vitamin B_6_	0.72	0.54	0.65	0.66
Vitamin B_12_	0.69	0.57	0.66	0.56
Folate	0.70	0.69	0.67	0.77
Pantothenic acid	0.71	0.76	0.61	0.69
Vitamin C	0.78	0.76	0.72	0.77

Daidzein	0.61	0.63	0.60	0.58
Genistein	0.61	0.63	0.60	0.58

**MEDIAN**	**0.70**	**0.63**	**0.61**	**0.58**

Abbreviations: SFA, saturated fatty acid; MUFA, monounsaturated fatty acid; PUFA, polyunsaturated fatty acid; Eq, equivalent.

For men, *r* ≥ 0.24 = *P* < 0.05, *r* ≥ 0.31 = *P* < 0.01, *r* ≥ 0.39 = *P* < 0.001. For women, *r* ≥ 0.23 = *P* < 0.05, *r* ≥ 0.30 = *P* < 0.01, *r* ≥ 0.38 = *P* ≤ 0.001.

**Supplementary Table 4.** Spearman rank correlation coefficients between 2 food frequency questionnaires, administered at an average interval 2.7 years, for estimated food-group intakes

	Men (*n* = 69)		Women (*n* = 75)	
		
	Crude	Energy- adjusted	Crude	Energy- adjusted
Cereals	0.63	0.69	0.49	0.55
Rice	0.64	0.62	0.65	0.63
Bread	0.73	0.70	0.55	0.60
Noodles	0.64	0.60	0.49	0.51
Other cereals	0.35	0.38	0.65	0.64
Potatoes and starches	0.60	0.60	0.65	0.60
Sugar	0.74	0.68	0.65	0.50
Pulses	0.45	0.51	0.65	0.56
Nuts and seeds	0.42	0.32	0.63	0.60
Vegetables	0.63	0.63	0.70	0.64
Green and yellow vegetables	0.64	0.58	0.61	0.51
White vegetables	0.69	0.62	0.69	0.62
Pickled vegetables	0.74	0.70	0.76	0.67
Cruciferous vegetables	0.65	0.60	0.50	0.46
Green, leafy vegetables	0.57	0.53	0.57	0.61
Yellow vegetables	0.60	0.55	0.60	0.46
Other vegetables	0.71	0.65	0.70	0.58
Fruits	0.70	0.67	0.63	0.69
Citrus fruit	0.67	0.61	0.62	0.66
Other fruit	0.66	0.64	0.61	0.55
Fungi	0.73	0.75	0.60	0.60
Algae	0.65	0.65	0.57	0.56
Fish and shellfish	0.62	0.39	0.70	0.62
Meats	0.69	0.57	0.54	0.52
Processed meat	0.67	0.62	0.71	0.70
Red meat	0.63	0.53	0.48	0.47
Poultry	0.54	0.36	0.50	0.49
Eggs	0.66	0.53	0.50	0.51
Milk and dairy products	0.73	0.69	0.61	0.52
High-fat milk	0.49	0.45	0.71	0.66
Low-fat milk	0.68	0.65	0.50	0.52
Fats and oils	0.63	0.53	0.63	0.51
Butter	0.57	0.45	0.63	0.55
Margarine and Oils	0.61	0.54	0.64	0.51
Confectionaries	0.63	0.60	0.63	0.64
Japanese	0.56	0.57	0.60	0.62
Western	0.66	0.62	0.60	0.55
Alcoholic beverages	0.86	0.86	0.76	0.68
Non-alcoholic beverages	0.61	0.61	0.68	0.63
Green tea	0.68	0.66	0.75	0.67
Coffee	0.75	0.69	0.80	0.76
Other beverage	0.45	0.48	0.56	0.52
Seasonings and spices	0.69	0.70	0.53	0.48

**MEDIAN**	**0.64**	**0.61**	**0.63**	**0.58**

For men, *r* ≥ 0.24 = *P* < 0.05, *r* ≥ 0.31 = *P* < 0.01, *r* ≥ 0.39 = *P* < 0.001. For women, *r* ≥ 0.23 = *P* < 0.05, *r* ≥ 0.30 = *P* < 0.01, *r* ≥ 0.38 = *P* ≤ 0.001.

**Supplementary Table 5.** Cumulative percentage contribution of the top 20 foods to energy intake, as assessed by 4-day diet record

Code	Food	kcal/day	Cumulative percent
Men (*n* = 69)			
1088	Rice, Paddy rice grain, Well-milled rice	422.9	18.6
1026	Breads, White table bread	61.1	21.3
16006	Fermented alcoholic beverages, Beer, pale	52.8	23.6
12004	Hen’s eggs, whole, raw	51.0	25.9
13003	Liquid milks, Ordinary liquid milk	49.5	28.0
1085	Rice, Paddy rice grain, Brown rice	45.8	30.1
14006	Fats and oils, Vegetable oil, blend	44.7	32.0
4046	*Natto* (Fermented soybean), *Itohiki-natto*	31.7	33.4
1048	Chinese noodles, Wet form, boiled	28.8	34.7
16015	Distilled alcoholic beverages, *Shochu*, 25% alcohol	25.2	35.8
13025	Yogurt, Whole milk, unsweetened	24.0	36.9
1087	Rice, Paddy rice grain, Under-milled rice	22.8	37.9
1039	*Udon*, Wet form, boiled	20.8	38.8
7107	Bananas, Raw fruit	19.2	39.6
11221	Chicken, Broiler meats, Thigh, with skin, raw	18.2	40.4
3003	Sugars, Soft sugars, White	17.6	41.2
1064	Macaroni, spaghetti, Dry form, boiled	16.2	41.9
2017	Potatoes, Tuber, raw	16.1	42.6
11123	Pork, large breeds, Loin, lean and fat, raw	16.1	43.3
4032	*Tofu* (soybean curd), *Momen-tofu*	15.7	44.0
Women (*n* = 74)			
1088	Rice, Paddy rice grain, Well-milled rice	286.0	15.5
1026	Breads, White table bread	67.9	19.2
13003	Liquid milks, Ordinary liquid milk	54.3	22.2
12004	Hen’s eggs, whole, raw	46.8	24.7
14006	Fats and oils, Vegetable oil, blend	36.4	26.7
1048	Chinese noodles, Wet form, boiled	28.6	28.2
1085	Rice, Paddy rice grain, Brown rice	21.1	29.4
4046	*Natto* (Fermented soybean), *Itohiki-natto*	20.9	30.5
1089	Rice, Paddy rice grain, Well-milled rice with germ	19.1	31.6
2017	Potatoes, Tuber, raw	17.9	32.5
4040	*Abura-age* (Fried thin slices of pressed *tofu*, soybean curd)	17.3	33.5
1039	*Udon*, Wet form, boiled	17.2	34.4
13025	Yogurt, Whole milk, unsweetened	16.6	35.3
7148	Apples, Raw fruit	15.8	36.2
16006	Fermented alcoholic beverages, Beer, pale	15.7	37.0
15098	Biscuits, soft, Western-style confectioneries	15.3	37.8
11221	Chicken, Broiler meats, Thigh, with skin, raw	14.8	38.6
14001	Fats and oils, Olive oil	14.4	39.4
1117	Glutinous rice products, Rice cake	14.1	40.2
7107	Bananas, Raw fruit	14.1	41.0
